# Supplementary material for: Calcite carbonate sinks low-density plastic debris in open oceans
Source: Nat Commun. 2024 Jun 6;15:4837. doi: 10.1038/s41467-024-49074-7 (PMC11156676; doi:10.1038/s41467-024-49074-7)
Supplement: Supplementary file 1 — Supplementary Information [file 41467_2024_49074_MOESM1_ESM.pdf]

Supplementary Materials for

Calcite carbonate sinks low-density plastic debris in open oceans

Xiang-Fei Sun[1,2], Yanxu Zhang[3], Meng-Yi Xie[1], Lei Mai[2], Eddy Y. Zeng[1,2,*]

[1] *School of Environment and Energy, South China University of Technology, Guangzhou 510006, China*

[2] *Southern Marine Science and Engineering Guangdong Laboratory (Zhuhai), Zhuhai 519080, China*

[3] *School of Atmospheric Sciences, Nanjing University, Nanjing 210023, China*

These authors contributed equally: Xiang-Fei Sun and Yanxu Zhang

* Corresponding author: Email: eddyzeng@scut.edu.cn (EYZ)

This PDF file includes

Figs. S1 to S3

Table S1 to S3

References

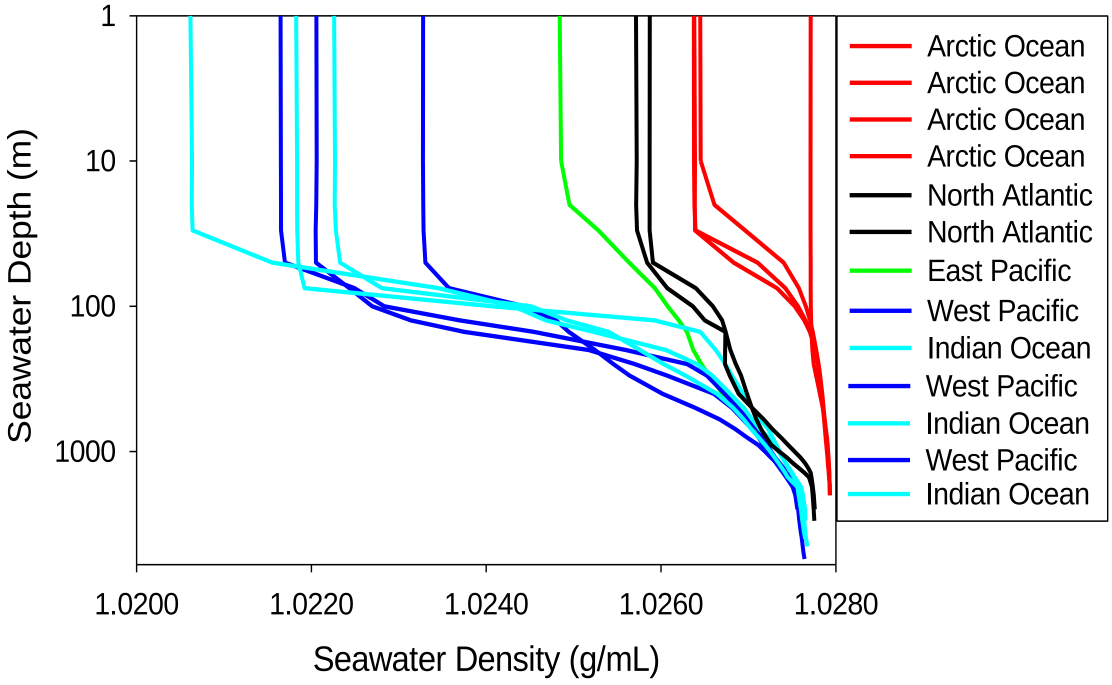

Figure S1. **The seawater density variation along seawater depth in all sampling sites using NOAA data**

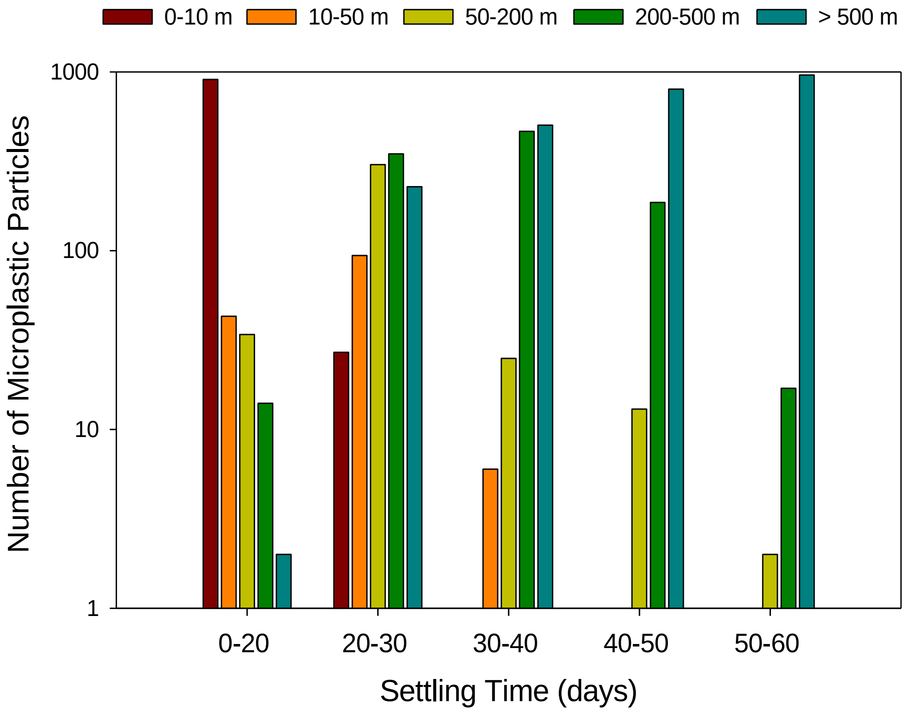

Figure S2. Vertical distribution patterns of plastic debris at selected time intervals
A group of plastic debris of various sizes and shapes was initially placed at the top of the seawater surface. At each time interval, the concentration of plastic debris at the selected depth interval was determined and plotted on the graph.

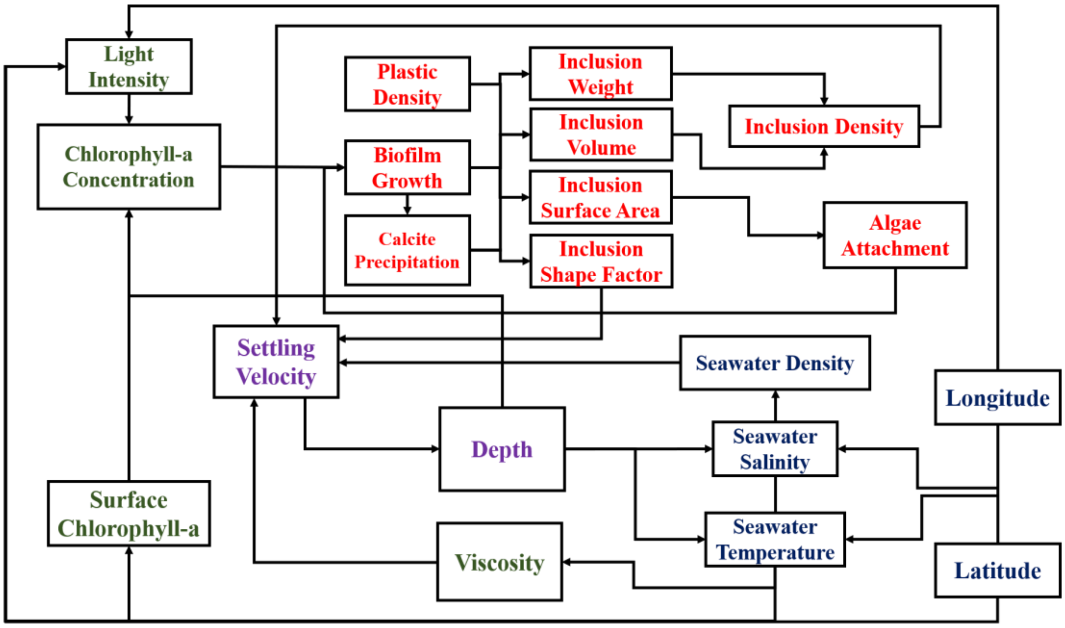

Figure S3. **General model structure and calculation flow**

Table S1. Shape similarity between observed and simulated plastic debris concentration in vertical seawater profiles

Site	Ocean	Location	Number of Measures	Shape Similarity	Reference
Moss Landing Harbor, USA	East Pacific Ocean	36.8°N, 122.3°W	10	0.722	[1]
SK-1*	West Pacific Ocean	23.2°N, 127.3°E	4	0.934	[2]
SK-2*	West Pacific Ocean	12.1°N, 141.8°E	9	0.944	[2]
SK-3*	West Pacific Ocean	14.8°N, 145.8°E	6	0.897	[2]
SY-1*	North Indian Ocean	13.5°N, 87.3°E	5	0.973	[2]
SY-2*	Tropical Indian Ocean	0.1°N, 87.3°E	5	0.829	[2]
SY-3*	South Indian Ocean	13.5°S, 87.3°E	5	0.865	[2]
Deploy-1*	North Atlantic Ocean	33.7°N, 40.5°W	8	0.764	[3]
Deploy-2*	North Atlantic Ocean	34.4°N, 36.2°W	8	0.884	[3]
EGIV	The Arctic Ocean	78.8°N, 2.8°E	4	0.999	[4]
N5	The Arctic Ocean	79.9°N, 3.1°W	4	0.999	[4]
HGIV	The Arctic Ocean	79.1°N, 4.2°W	4	0.999	[4]
HGIX	The Arctic Ocean	79.1°N, 2.8°W	4	0.646	[4]

Notes: * represents the best-estimated GPS of the given location.

Table S2. Parameter symbols, values, and units in the model program

Category	Symbol	Name	Quantity	Unit
1	den_A	Algae Density	1380	kg m^{-3}
2	V_A	Algae Volume	1.00E-15	m^{3}
3	m_A	Mortality Ratio	0.39	d^{-1}
4	R_20	Respiration Rate	0.1	d^{-1}
5	k	Boltzmann Constant	1.03E-13	m^{2} kg d^{-2} K^{-1}
6	Q_10	Temperature Coefficient Respiration	2	-
7	u_max	Maximum Growth Rate of Algae	1.85	d^{-1}
8	alpha	Initial Slope	0.12	d^{-1}
9	T_min	Minimum Temperature for Algae Growth	0.2	℃
10	T_max	Maximum Temperature for Algae Growth	33.3	℃
11	T_opt	Optimal Temperature for Algae Growth	26.7	℃
12	I_opt	Optimal Light Intensity at noon	1.75E+13	μE m^{-2} d^{-1}
13	o_w	Extinction coefficient water	0.2	m^{-1}
14	o_p	Extinction coefficient algae	0.02	m^{-1} mg chl L^{-1}
15	I_m	Surface Light Intensity at Noon	1.20E+08	μE m^{-2} d^{-1}
16	shear	Shear	1.70E+05	d^{-1}
17	phy_att	Collision Ratio	0.25	d^{-1}
18	Ca_g	Calcite Precipitation Rate	0.5	kg d^{-1} m^{-2}
19	r_pl	The radius of Plastic debris in a Sphere Shape	Variable	m
20	den_pl	Plastic debris Density	Variable	kg m^{-3}
21	l_pl	Fiber Length	Variable	m
22	t_pl	Film Thickness	Variable	m
23	r_fiber	Fiber Cross-section Radius	Variable	m
24	r_film	Film Radius	Variable	m
25	shape_f	Shape Factor	Variable	-
26	Wt	Settling Speed	Variable	m day^{-1}
27	Re	Reynolds Number	Variable	-
28	w_v	Seawater Viscosity	Variable	kg m^{-1} day^{-1}
29	den_f	Seawater Density at Given Location and Depth	Variable	kg m^{-3}
30	pore_T	Seawater Temperature at Given Location and Depth	Variable	℃
31	pore_salinity	Seawater Salinity at Given Location and Depth	Variable	ppt
32	a1	Parameters Viscosity Profile	1.57E-01	-
33	a2	Parameters Viscosity Profile	6.50E+01	-
34	a3	Parameters Viscosity Profile	-9.13E+01	-
35	a4	Parameters Viscosity Profile	4.28E-05	-
36	a5	Parameters Viscosity Profile	1.54E+00	-
37	a6	Parameters Viscosity Profile	2.00E-02	-
38	a7	Parameters Viscosity Profile	-9.52E-05	-
39	a8	Parameters Viscosity Profile	7.97E+00	-
40	a9	Parameters Viscosity Profile	-7.56E-02	-
41	a10	Parameters Viscosity Profile	4.72E-04	-
42	A	Parameters Gravity Profile	0.0053024	-
43	B	Parameters Gravity Profile	0.0000058	-

44	C	Parameters Gravity Profile	3.09E-06	-
45	b1	Parameters Density Profile	8.02E+02	-
46	b2	Parameters Density Profile	-2.001	-
47	b3	Parameters Density Profile	1.68E-02	-
48	b4	Parameters Density Profile	-3.06E-05	-
49	b5	Parameters Density Profile	-1.61E-05	-
50	c1	Parameters Density Profile	1.00E+03	-
51	c2	Parameters Density Profile	2.03E-02	-
52	c3	Parameters Density Profile	-6.16E-03	-
53	c4	Parameters Density Profile	2.26E-05	-
54	c5	Parameters Density Profile	-4.66E-08	-
55	chl_a	Surface Chlorophyll-a Concentration	Variable	mg m^{-3}
56	z	Ocean Depth	Variable	m
57	C_conc	Carbon Concentration	Variable	mg m^{-3}
58	A_conc	Algae Concentration	Variable	cell m^{-3}
59	L_z	Light Intensity at Given Depth z	Variable	μE m^{-2} day^{-1}
60	S_pl	Surface Area of Original Plastic Debris	Variable	m^2
61	B_g	Biofilm Growth Rate	Variable	cell m^{-2} day^{-1}
62	V_bf	Accumulated Volume of Algae in the Biofilm	Variable	m^3
63	V_M	Accumulated Volume of Minerals in the Biofilm	Variable	m^3
64	m_b	Accumulated Mass of Calcite in the Biofilm	Variable	kg
65	V_T	Total Volume of Plastic debris Inclusion	Variable	m^3
66	d_ESD	Equivalent Spherical Diameter	Variable	m
67	Theta	Sphericity	Variable	-
68	X	Circularity	Variable	-
69	in_den	Plastic debris Inclusion Density	Variable	kg m^{-3}

Table S3. Parameter symbols, values, and units for Chlorophyll-a Estimation[5,6]

Symbol	Name	Unit	0-0.04	0.04-0.08	0.08-0.12	0.12-0.2	0.2-0.3	0.3-0.4	0.4-0.8	0.8-2.2	2.2-4
Cb	Normalized Surface Value	-	0.471	0.533	0.428	0.57	0.611	0.39	0.569	0.835	0.188
s	Normalized Slop	-	0.135	0.172	0.002	0.173	0.214	0.108	0.183	0.298	0
C_max	Normalized peak Concentration	-	1.572	1.194	1.015	0.766	0.676	0.788	0.608	0.382	0.885
Z_max	Depth of the Peak	m	115.41	92.01	82.36	65.28	46.61	33.03	24.59	20.38	9.87
delta_z	Width of the Peak	m	46.81	43.46	57.33	47	37.89	43.18	40.47	24.88	28.21
mean_Chl_a	Average Chl_*a* Concentration	mg m^{-3}	0.091	0.151	0.185	0.25	0.338	0.41	0.578	1.206	2.95
norm_Z_max	Normalized Depth of the Peak	-	0.969	0.921	0.905	0.814	0.663	0.521	0.452	0.512	0.378
euphotic depth	Euphotic Zone Depth	m	119	100	91	80	70	63	54	40	26

Supplementary References

1 Choy, C. A., Robison, B. H., Gagne, T. O., Erwin, B., Firl, E., Halden, R. U., Hamilton, J. A., Katija, K., Lisin, S. E., Rolsky, C. & S. Van Houtan, K. The vertical distribution and biological transport of marine microplastics across the epipelagic and mesopelagic water column. *Sci. Rep.* **9**, 7843, doi:10.1038/s41598-019-44117-2 (2019).

2 Li, D., Liu, K., Li, C., Peng, G., Andrady, A. L., Wu, T., Zhang, Z., Wang, X., Song, Z., Zong, C., Zhang, F., Wei, N., Bai, M., Zhu, L., Xu, J., Wu, H., Wang, L., Chang, S. & Zhu, W. Profiling the vertical transport of microplastics in the West Pacific Ocean and the East Indian Ocean with a novel in situ filtration technique. *Environ. Sci. Technol.* **54**, 12979-12988, doi:10.1021/acs.est.0c02374 (2020).

3 Galgani, L., Goßmann, I., Scholz-Böttcher, B., Jiang, X., Liu, Z., Scheidemann, L., Schlundt, C. & Engel, A. Hitchhiking into the deep: How microplastic particles are exported through the biological carbon pump in the north Atlantic Ocean. *Environ. Sci. Technol.* **56**, 15638-15649, doi:10.1021/acs.est.2c04712 (2022).

4 Tekman, M. B., Wekerle, C., Lorenz, C., Primpke, S., Hasemann, C., Gerdts, G. & Bergmann, M. Tying up loose ends of microplastic pollution in the Arctic: Distribution from the sea surface through the water column to deep-sea sediments at the HAUSGARTEN observatory. *Environ. Sci. Technol.* **54**, 4079-4090, doi:10.1021/acs.est.9b06981 (2020).

5 Uitz, J., Claustre, H., Morel, A. & Hooker, S. B. Vertical distribution of phytoplankton communities in open ocean: An assessment based on surface chlorophyll. *J. Geophys. Res. Oceans* **111**, doi:https://doi.org/10.1029/2005JC003207 (2006).

6 Ardyna, M., Babin, M., Gosselin, M., Devred, E., Bélanger, S., Matsuoka, A. & Tremblay, J. É. Parameterization of vertical chlorophyll-a in the Arctic Ocean: impact of the subsurface chlorophyll maximum on regional, seasonal, and annual primary production estimates. *Biogeosciences* **10**, 4383-4404, doi:10.5194/bg-10-4383-2013 (2013).
